# Biocompatible Porous Tantalum Metal Plates in the Treatment of Tibial Fracture

**DOI:** 10.1111/os.12432

**Published:** 2019-03-18

**Authors:** De‐wei Zhao, Zhi‐jie Ma, Tie‐nan Wang, Bao‐yi Liu

**Affiliations:** ^1^ Department of Orthopedics Affiliated Zhongshan Hospital of Dalian University Dalian China

**Keywords:** Bone plate, Nonunion, Porous tantalum metal, Tibial fracture

## Abstract

Fractures of the tibia represent a common class of injuries in orthopedics. The blood supply to the tibia is poor due to the small subcutaneous muscle tissues inside. Consequently, the tibia is prone to delayed fracture healing and nonunion of the fracture after surgery. In this case, we used porous tantalum metal plate to treat nonunion of a tibial fracture and achieved satisfactory therapeutic effects. For the first time in the field, we used 3D printing technology to fabricate porous tantalum metal plates for the treatment of tibial fractures. The resulting porous tantalum metal exhibited excellent mechanical and biological properties, and improved the therapeutic effects for the treatment of a tibial fracture nonunion. Porous tantalum metal plates have great application potential as a new implant material for internal fixation.

## Introduction

Tibial fractures are generally caused by high‐energy injuries, and the incidence rate is 16.9/100 000^1^. Moreover, nonunion and malunion account for 2%–12% of all fractures of the tibia^2^. Based on different injury mechanisms, the nonunion and malunion of tibial fractures can be categorized into hypertrophic, malnutrition, atrophy, infection and synovial pseudoarticular formation^3^, ^4^. In recent years, researchers worldwide have extensively studied the mechanism and treatment of different types of nonunion and have developed various new ideas and methods, but the primary concern is still the local stability and maintenance of proper blood supply^5^. Although various surgical treatment and repair methods of nonunion and malunion of tibial fractures have been rapidly developed, the optimal treatment solution for these fractures remains controversial. We admitted a patient who lived at an altitude of 4600 m and suffered from nonunion of a tibial fracture that failed to heal after three operations. In this case, we used a porous tantalum metal plate to treat nonunion of the tibial fracture and achieved satisfactory therapeutic effects.

## Case Report

**Figure 1:**
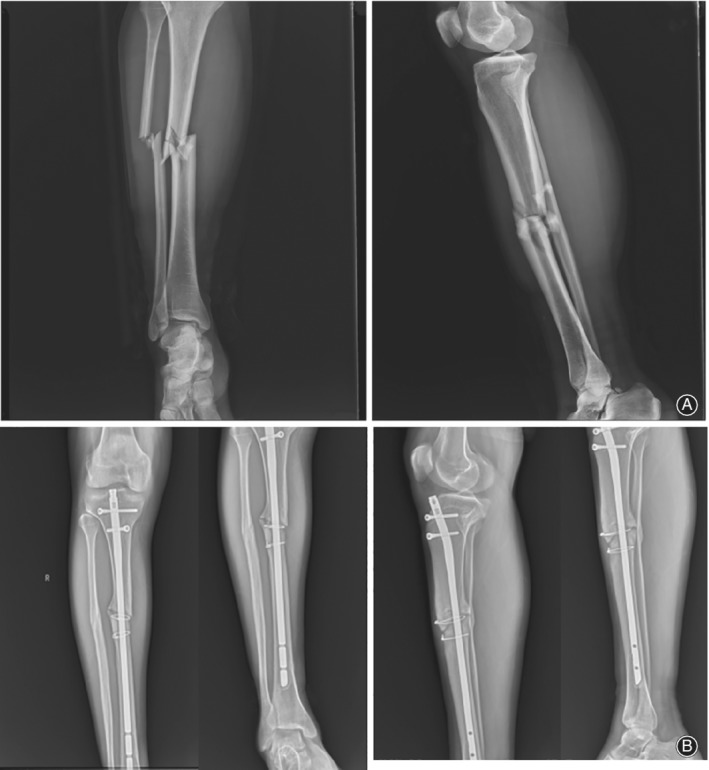
(A) The lateral radiograph of the tibiofibula shows the comminuted fracture of the right tibiofibular; the fracture was obviously displaced. (B) Three years after surgery, the lateral radiograph of the tibiofibula shows the nonunion of the right tibia; the fracture line is clear and sclerotic bone formation can be seen at the fracture end.

**Figure 2:**
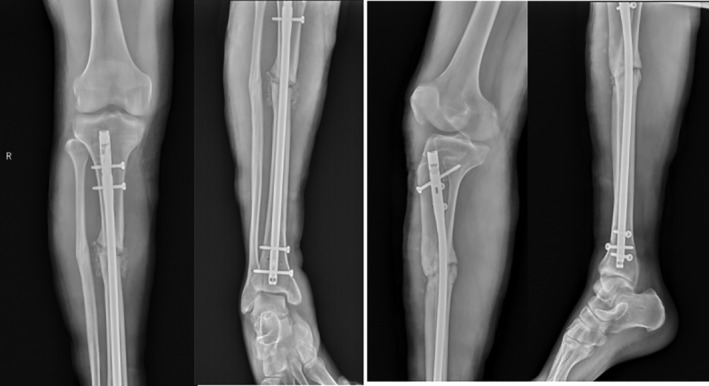
The patient underwent surgery again, replacing the large intramedullary nail and taking the right iliac bone for bone grafting. Three months later, the upper end of the intramedullary nail screw was removed, to increase micro‐power, eliminate the stress shielding effect and promote fracture healing.

**Figure 3:**
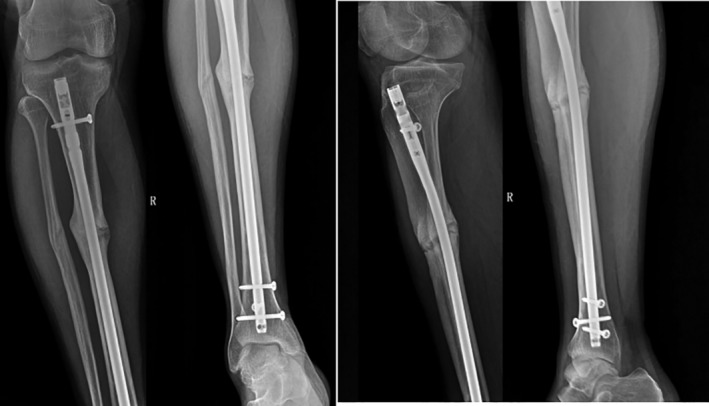
Two years after the third operation, the right tibial fracture remained unhealed, the fracture line still existed, there was tenderness around the medial tibial fractur, and there was pain in the affected limb during weight bearing.

The patient, a 30‐year‐old Tibetan man, was treated in the Lhasa Hospital for right tibiofibular fracture December 2012 (Fig. 1). After the swelling subsided, he underwent right tibia fracture intramedullary internal fixation. Two weeks after the operation, the patient was able to walk with double crutches without load, and was able to walk without crutches and with some load 3 months after the operation. However, the patient complained of pain in his right lower extremity, especially when moving downhill or down stairs, and experienced a limping gait and obvious tenderness at the fracture end. Three years after originally presenting at Lhasa Hospital, the patient was experiencing persistent pain in the right lower extremity, and his daily activities were severely affected. Thus, he was re‐diagnosed with nonunion of the right tibial fracture after internal fixation (Fig. 1). The main causes of the nonunion of the fracture were considered to be the excessively thin intramedullary nails and unstable fixation of the fracture. The operation was repeated with replacement of the crude intramedullary nails and grafting of iliac bone (Fig. 2). Three months after the operation, the tibial fracture had failed to heal, and further surgical treatment was performed. The proximal locking screw of the intramedullary nail was removed with an expectation to eliminate the stress shielding effect and to promote fracture healing by increasing the microdynamic force while retaining the static interlocking nail. Five months after the operation, the patient abandoned the crutches and was able to walk with a heavy load. Nevertheless, 2 years after the operation, active pain of the right leg persisted with some tenderness around the fracture, especially when moving downhill or down stairs. August 2018 the patient was admitted to our hospital. Physical examination revealed that two old incision healing scars, approximately 2 cm in length, were present at the proximal and distal ends of the right tibia. The middle section showed a scar from an approximately 10‐cm incision; pigmentation was found around the incision and tenderness was experienced around the fracture. Imaging results revealed a nonunion of the right tibial fracture, and the admission diagnosis was nonunion of the right tibial fracture after surgery (Fig. 3).

Two days after admission, “right tibial locking intramedullary nail removal, open reduction and porous tantalum metal plate fixation” was performed. After removal of intramedullary nails during the operation, nonunion of the tibial fracture was corrected and osteoporosis of the fracture end. Osteosclerosis of the fracture was observed, and the sclerotic bones as well as part of the hyperplastic epiphysis were removed; the marrow was reamed at the fracture to keep the medullary cavity open. The resected osteophytes were implanted into the fracture, followed by fixation with a porous tantalum plate.

**Figure 4:**
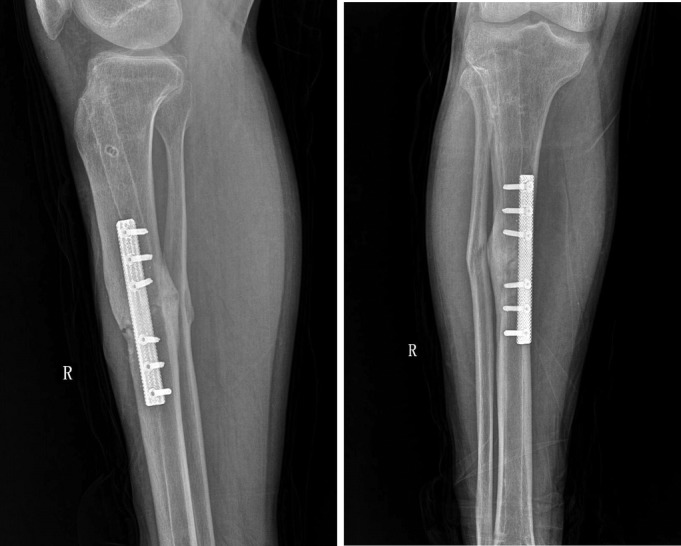
Five months after the fixation of the porous tantalum metal plate, the fracture gradually healed and the patient could move and work normally.

After the operation, the affected limbs were fixed with plaster. Ankle joint activity training was initiated on the first day after the operation, and knee joint activity training began 2 weeks after the operation. The patient was able to walk with double crutches without load 4 weeks after the operation and could perform normal activities 12 weeks after the operation without pain in the right limb. However, slight tenderness was still experienced around the fracture. Five months after the surgery, the right tibial fracture had healed, based on imaging examination, and the tenderness around the fracture had disappeared; the patient was able to work normally (Fig. 4).

## Discussion

Poor blood supply represents the main cause of delayed union and nonunion of tibial fractures^6^. The blood supply of the tibial shaft consists of the nourishing arterial system, the periosteal vascular system, and the epiphyseal vasculature system. The rate of delayed union and nonunion of fractures can be over three times as high as that of other vascular injuries^7^. The tibia nourish artery is one of the three major blood vessels and is mainly derived from the posterior tibial artery. It passes through the proximal end of the posterior tibial muscle group and enters the cortical bone in the trophoblast of the lateral aspect of the middle part of the tibia. The tibia nourish artery is highly vulnerable to injuries, which can result in significant reduction in blood supply to the bones, thereby affecting the healing of fractures. The incidence of nonunion of humeral shaft fractures in the clinic is 5%, the incidence of delayed healing of open tibiofibular fracture is 6.80%, and the incidence of delayed healing of one‐third of the middle tibia is as high as 92.40%, creating great difficulties for clinical treatment^8^. At present, there are various types of internal fixation methods for the treatment of tibial fracture malunion and nonunion. The treatment options include steel plates, intramedullary nails, external fixators, bone grafting, and a combination of these techniques.

As early as 1991, Mohsen *et al.* 9 used a single external fixator to treat the delayed union of a tibial shaft fracture. The IIizarov external fixator, an effective surgical method, can not only treat a nonunion of the tibial fracture but also treat a complex open fracture of the tibia. Although the external fixation technology has achieved great stability of the fracture end, the healing process can be delayed by excessively strong external fixators^10^. Currently, the compression locking plate (LCP) is a common surgical technique for the treatment of malunion and nonunion of tibial fractures. El‐Rosasy *et al.* 5 showed that tibial fractures with angulation deformity less than 35° and limb length difference less than 2.4 cm after correction could be treated with internal fixation and compression locking plates; using this method resulted in few complications and, therefore, it proved an ideal internal fixation technique for promoting bone healing. However, the drawbacks of LCP are that it cannot provide adequate stability and that it is associated with poor postoperative treatment for patients with large bone defects and nonunion, resulting in further malunion or even nonunion of fractures^11^.

Since the concept of minimally invasive closed reduction was proposed, interlocking intramedullary nails (IMN) have been widely used in long bone fractures^12^, ^13^. A prospective study by Naik *et al.* 13 showed that IMN can reduce economic burden and medical costs for society to a greater extent than the minimally invasive proximal tibial plate fixation (PTP). Titanium has superior biocompatibility to stainless steel, and the use of titanium rod intramedullary nails inhibits the foreign body reactivity of soft tissues and reduces the chance of infection. The research and development as well as the clinical applications of dynamic intramedullary nails have become major focuses in the field. The technology can enhance the micro‐dynamics while retaining the static internal fixation of the static locking nails, eliminate the stress shielding effect, promote fracture healing, and reduce the occurrence of fracture nonunion and malunion. With the widespread application of intramedullary nails for the treatment of long bone fractures, their disadvantages of causing fracture nonunion or delayed union have gradually manifested. Meanwhile, remedullary reaming can result in additional vascular damage and cortical necrosis of the periosteum, leading to nonunion of the fracture. Even in the absence of reaming, delayed union of intramedullary nails may occur due to failure of locking^14^.

In this case, the tibial fracture failed to heal even after interlocking intramedullary nail fixation was performed twice, exerting tremendous psychological and economic burden on the patient. We wondered if there was a fixation method that could not only promote fracture healing but also alleviate the pain of the patient. In recent years, porous tantalum metal has attracted considerable attention among material scientists and clinicians because of its excellent corrosion resistance and outstanding biocompatibility. In clinical observations, porous tantalum bone implanted devices exhibited excellent biocompatibility, and the porous tantalum and bone tissues were found to form strong binding with excellent long‐term stability^15^, ^16^, ^17^.

For the first time, we used 3D printing technology to prepare porous tantalum metal plates for the treatment of tibial fractures. The main advantages include, first, that that porous tantalum metal plate features a lightweight design with a porosity of up to 80%. This design not only ensures the mechanical strength of the plate but also reduces the weight of the implant and increases the comfort of the patients. Second, the modulus of elasticity of the porous tantalum metal plate is 1.5–10 GPa, which is between the values of the cancellous bone and the cortical bone. This feature effectively reduces stress shielding and avoids fracture of the implant. Third, the porous structure of the porous tantalum metal plate can affect the proliferation of osteoblasts, and the rough surfaces can readily absorb macromolecules, thereby influencing cell proliferation, adhesion, and osteogenesis, and eventually promoting fracture healing. Finally, the molecular structure of the porous tantalum metal plate is a 3D connected network of dodecahedral complexes. After implantation, this type of structure facilitates the regeneration, reconstruction and growth of bone tissues, enhances the ability of the device to connect with bone tissues, and increases the long‐term biological stability of the implanted device. Five months after implantation of the porous tantalum metal plate, imaging examination revealed that the fracture line had disappeared, and the tibial fracture had healed. The patient no longer complained of tenderness around the fracture and was able to perform normal work. The treatment effect was satisfactory.

In summary, while the best surgical method for the treatment of tibial fracture malunion or nonunion remains controversial, a surgical approach is still the main means of treating tibial nonunion. Biomechanical and biocompatibility factors that cause delayed union should be considered in the treatment. In the current study, porous tantalum metal plate exhibited satisfactory therapeutic effects due to its excellent mechanical properties and exceptional biocompatibility. However, the investigation of long‐term efficacy requires multi‐center cooperation and a larger amount of sample data.
